# Supplementary material for: Deletion of wfs1 Impairs Oligodendrocyte Precursor Cells Dorsal Distribution and Myelination Through the wfs1-hmgcs1 Axis in Zebrafish
Source: Biology (Basel). 2026 Aug 16;15(16):1402. doi: 10.3390/biology15161402 (PMC13510151; doi:10.3390/biology15161402)
Supplement: Supplementary file 1 [file biology-15-01402-s001.zip › File S1-Transcriptome sequencing data and differentially expressed genes (DEGs) in wfs1-- zebrafish at 5 dpf/2.KEGG/wfs1KOvsWT/GSEA/wfs1KO/FATTY_ACID_ELONGATION/FATTY_ACID_ELONGATION(DRE00062).html]

Details for gene set FATTY\_ACID\_ELONGATION(DRE00062)[GSEA]

|  || Dataset | deg.sample |
| Phenotype | sample.cls |
| Upregulated in class | wfs1KO |
| GeneSet | FATTY\_ACID\_ELONGATION(DRE00062) |
| Enrichment Score (ES) | 0.3781792 |
| Normalized Enrichment Score (NES) | 1.3383902 |
| Nominal p-value | 0.15667312 |
| FDR q-value | 0.25757027 |
| FWER p-Value | 0.931 |
Table: GSEA Results Summary

  

Fig 1: Enrichment plot: FATTY\_ACID\_ELONGATION(DRE00062)      
 Profile of the Running ES Score & Positions of GeneSet Members on the Rank Ordered List

  

| PROBE | DESCRIPTION (from dataset) | GENE SYMBOL | GENE\_TITLE | RANK IN GENE LIST | RANK METRIC SCORE | RUNNING ES | CORE ENRICHMENT || 1 | 393441 | hacd3 |  |  | 1017 | 2.075 | 0.0388 | Yes |
| 2 | 553401 | hadhaa |  |  | 1608 | 1.593 | 0.0746 | Yes |
| 3 | 554145 | elovl8b |  |  | 2103 | 1.324 | 0.1042 | Yes |
| 4 | 334217\_1 | elovl7a |  |  | 2141 | 1.310 | 0.1477 | Yes |
| 5 | 553440 | acot20 |  |  | 2200 | 1.287 | 0.1898 | Yes |
| 6 | 393135 | zgc:55621 |  |  | 2338 | 1.233 | 0.2275 | Yes |
| 7 | 449816 | elovl1a |  |  | 2673 | 1.120 | 0.2552 | Yes |
| 8 | 327274 | elovl7b |  |  | 2805 | 1.083 | 0.2880 | Yes |
| 9 | 368367\_1 | hsd20b2 |  |  | 2911 | 1.057 | 0.3208 | Yes |
| 10 | 393657 | zgc:66024 |  |  | 3724 | 0.885 | 0.3254 | Yes |
| 11 | 678614 | elovl2 |  |  | 3951 | 0.839 | 0.3470 | Yes |
| 12 | 406830 | zgc:55413 |  |  | 4240 | 0.787 | 0.3647 | Yes |
| 13 | 335732 | elovl4b |  |  | 4729 | 0.714 | 0.3738 | Yes |
| 14 | 447878 | acot7 |  |  | 5292 | 0.647 | 0.3782 | Yes |
| 15 | 336606 | hadhb |  |  | 6664 | 0.523 | 0.3530 | No |
| 16 | 562047 | acot21 |  |  | 7621 | 0.445 | 0.3381 | No |
| 17 | 368367 | hsd20b2 |  |  | 8757 | 0.384 | 0.3156 | No |
| 18 | 550550 | mecr |  |  | 9422 | 0.339 | 0.3063 | No |
| 19 | 793834 | hadhab |  |  | 10077 | 0.298 | 0.2959 | No |
| 20 | 405845 | zgc:77118 |  |  | 10210 | 0.290 | 0.3016 | No |
| 21 | 406325 | acaa2 |  |  | 11783 | 0.202 | 0.2591 | No |
| 22 | 100150288 | elovl6l |  |  | 12263 | 0.176 | 0.2501 | No |
| 23 | 322626 | hsd17b12b |  |  | 12386 | 0.170 | 0.2521 | No |
| 24 | 566085 | tecra |  |  | 12679 | 0.155 | 0.2482 | No |
| 25 | 406725 | elovl1b |  |  | 13166 | 0.132 | 0.2374 | No |
| 26 | 327417 | hsd17b12a |  |  | 13341 | 0.124 | 0.2362 | No |
| 27 | 100126133 | ppt2 |  |  | 13345 | 0.123 | 0.2403 | No |
| 28 | 553326 | hacd1 |  |  | 16399 | -0.014 | 0.1449 | No |
| 29 | 767653 | elovl8a |  |  | 17124 | -0.037 | 0.1234 | No |
| 30 | 445121 | hadh |  |  | 18086 | -0.080 | 0.0960 | No |
| 31 | 368912 | echs1 |  |  | 18310 | -0.089 | 0.0920 | No |
| 32 | 317738 | elovl6 |  |  | 18840 | -0.116 | 0.0794 | No |
| 33 | 791152 | them4 |  |  | 19201 | -0.131 | 0.0725 | No |
| 34 | 798606 | tecrl2a |  |  | 22001 | -0.267 | -0.0063 | No |
| 35 | 561303 | si:ch73-41h24.1 |  |  | 22757 | -0.311 | -0.0194 | No |
| 36 | 101884755 | LOC101884755 |  |  | 22823 | -0.315 | -0.0107 | No |
| 37 | 326954 | tecrb |  |  | 23996 | -0.391 | -0.0341 | No |
| 38 | 450002 | tecrl2b |  |  | 24206 | -0.407 | -0.0268 | No |
| 39 | 492778 | acot19 |  |  | 24610 | -0.422 | -0.0251 | No |
| 40 | 393425 | elovl5 |  |  | 26119 | -0.539 | -0.0541 | No |
| 41 | 393769 | elovl4a |  |  | 26365 | -0.563 | -0.0426 | No |
| 42 | 317738\_1 | elovl6 |  |  | 28857 | -0.889 | -0.0905 | No |
| 43 | 393441\_1 | hacd3 |  |  | 29812 | -1.168 | -0.0806 | No |
| 44 | 334121 | hacd2 |  |  | 29838 | -1.181 | -0.0411 | No |
| 45 | 406648 | ppt1 |  |  | 29919 | -1.210 | -0.0024 | No |
| 46 | 334217 | elovl7a |  |  | 30920 | -1.885 | 0.0305 | No |
Table: GSEA details [plain text format]

  

Fig 2: FATTY\_ACID\_ELONGATION(DRE00062)      
 Blue-Pink O' Gram in the Space of the Analyzed GeneSet

  

Fig 3: FATTY\_ACID\_ELONGATION(DRE00062): Random ES distribution      
 Gene set null distribution of ES for **FATTY\_ACID\_ELONGATION(DRE00062)**
